# Plant diversity is closely related to the density of zokor mounds in three alpine rangelands on the Tibetan Plateau

**DOI:** 10.7717/peerj.6921

**Published:** 2019-05-13

**Authors:** Yujie Niu, Jianwei Zhou, Siwei Yang, Bin Chu, Huimin Zhu, Bo Zhang, Qiangen Fang, Zhuangsheng Tang, Limin Hua

**Affiliations:** 1 College of Grassland Science/Key Laboratory of Grassland Ecosystem of the Ministry of Education, Gansu Agricultural University, Lanzhou, Gansu, China; 2 Institute of Animal and Veterinary Science, Bijie, Guizhou, China

**Keywords:** Alpine rangeland, Animal disturbance, Zokor mounds, Plant diversity

## Abstract

**Background:**

Plateau zokor (*Myospalax baileyi*) is a subterranean rodent endemic to the Tibetan Plateau. This species has been generally viewed as a pest in China due to the competition for food with livestock and also causing soil erosion. As a result, plateau zokor has been the target of widespread poisoning or trapping campaigns designed to control or eliminate it since 1970s. But there is little research on the effect of plateau zokor on plant diversity in alpine rangelands. Therefore, objectively evaluating the positive effects of the plateau zokors disturbance on their living environment and plant communities is of great significance to understand the function of plateau zokor in alpine ecosystem.

**Methods:**

Here, we selected three rangelands (alpine meadow, alpine steppe and alpine shrub meadow) in which plateau zokors are typically distributed on the Tibetan Plateau, and five zokor mound density gradients were selected in each rangeland type to study the effects of the mounds on soil moisture and temperature related to plant species diversity.

**Results:**

The results showed that, with the mound density increasing, the soil temperature decreased significantly in all three rangeland types, and the soil moisture significantly increased in all three rangeland types. In the alpine meadow, both the plant diversity and cumulative species richness increased significantly with increasing mound density. The increase in broad-leaved forbs is the main reason for the increase of plant diversity in the alpine meadow disturbed by zokor mounds. In the alpine steppe, the plant diversity decreased significantly with increasing mound density, while the cumulative species richness initially decreased and then increased. In the alpine shrub meadow, the plant diversity first increased and then decreased with increasing mound density as did the cumulative species richness. In conclusion, plateau zokor mounds dominated the distribution of soil moisture and temperature and significantly affected plant diversity in these three rangelands on Tibetan Plateau; the results further deepen our understanding toward a co-evolved process.

## Introduction

Disturbance, both anthropogenic and natural, has increasingly attracted attention from ecologists in the last decades (Dornelas, 2010; Mackey & Currie, 2001; Martorell & Peters, 2009; Sousa, 1984). Disturbance is an important aspect in the process of natural selection and the evolution in biology, because it alters the environment in which every living being plays its significant functions (Battisti, Poeta & Fanelli, 2016a). Disturbance, to an extent, is able to characterize the structure and function of ecosystems composed of stability, diversity, resistance, and resilience, and is the fundamental to explain the potential mechanism of species coexistence in communities (Sousa, 1984). The role of disturbance in determining biodiversity on natural ecosystem has drawn close attention (Dornelas, 2010). Effects of disturbance on biological diversity have been studied in a wide range of ecosystems from tropical community to tundra communities, a range of organisms extending from smallest microorganisms to larger mammals (Battisti, Poeta & Fanelli, 2016b). The significant distinction of disturbance can be classified into two different categories: abiotic disturbances due to physicochemical and mechanical agents, and biotic disturbances due to the action of living organisms (Battisti, Poeta & Fanelli, 2016a). Abiotic disturbances derived from non-living things and include processes and factors created by different forms of energy, such as light energy, mechanical energy and chemical energy (Battisti, Poeta & Fanelli, 2016a). Furthermore, the abiotic disturbances in ecosystem are usually rapid, and have major impacts with positive or negative. But the biotic disturbances include all the events created by living organisms (Battisti, Poeta & Fanelli, 2016a; Gibson, 1989). The biotic disturbances in ecosystem are usually a slow process with co-evolution, especially for animal disturbance. The animal-induced disturbances are very common in natural ecosystems and are related to the different functions accomplished by living organisms over their life cycles (Brawn, Robinson & Thompson, 2001; Hobbs & Huenneke, 1992; Martorell & Peters, 2009). Through long-term adaptation, animals form a balanced relationship of interdependence and mutual restraint with their surrounding biotic communities and inorganic environments. So, the study of animal disturbance on environment can help deepen our understanding toward co-evolved processes.

Subterranean rodents occur in large populations and have wide distributions in the world, and they play a major role in the structure and function of ecosystem (Zhang, 2007). Across the globe, at least 250 extant rodent species (38 genera, six families) spend most of their lives in self-constructed burrows (Begall, Burda & Schleich, 2007). Rodents disturbances can be at least classified into four primary categories: tunnel digging, foraging, feces and urine deposition (Battisti, Poeta & Fanelli, 2016a). Tunnel digging is the main activity performed by all those species to further affect the ecosystems where they live (Zhang & Liu, 2003). Digging and mound-building by subterranean rodents can exert considerable disturbances on the topsoil and may further affect plant composition and diversity of communities. Plateau zokors (*Myospalax baileyi*), a species unique to the alpine rangeland ecosystems of Tibetan Plateau (Wang et al., 2008), live solitarily underground in extensive burrows that they use for foraging, mating and nurturing. Plateau zokor dig the tunnels and push the soil inside the tunnels to form mounds of excavated material on the ground. The mounds are mainly distributed in alpine meadow, alpine shrub meadow and alpine steppe on the Tibetan Plateau (Zhang et al., 2014). Plateau zokors rapidly dig burrows in the soil, and each individual creates an average of 40–80 mounds per year. On average one mound with a mean volume of 0.007 m^3^ is formed by a single zokor everyday (Zhang, 2007). Although much research in population biology of plateau zokors have emerged over many years, the relationships between zokors and plants community, especially for the effects of zokor disturbance on the structure and diversity of pant communities, have been poorly recognized (Zhang, Zhang & Liu, 2003).

In China, plateau zokors have been considered as a pest due to the competition for food with livestock and also causing soil erosion (Zhang & Liu, 2003; Zhang, Zhang & Liu, 2003). The Ministry of Agriculture has approved the projects that kill plateau zokor. Widespread mechanical or poisoning activities have been implemented to kill the pest since 1970s (Fig. S1). Only in 2014, extermination work had been carried out on 31,151 km^2^ to kill plateau zokor on alpine rangeland (Gan, 2014). However, the positive ecological effects of zokor disturbance on rangeland has been completely neglected. The mounds of excavated soil may overlay plants but provide colonization opportunities for plant species and induce plant community succession. Moreover, the zokor mounds created greater micro-habitat heterogeneity of plant species. The heterogeneity can be defined as the different components of an ecosystem which is linked to the time (temporal dynamic) and space (spatial heterogeneity). The environments which are highly heterogeneous, make a great range of ecological conditions of the living spaces around the biologic organisms, allow the presence of more niches, and as a result more species can exist (Battisti, Poeta & Fanelli, 2016a; Begon, Townsend & Harper, 2006). The plateau zokor disturbance with constantly digging below ground and the mound-building activities on soil surface, modify the parts of soil in different layers thus facilitating the migration of air, water, solution and other organisms (Smith & Foggin, 1999; Zhang, Zhang & Liu, 2003). The mounds areas can provide an ecological opportunity which is conducive to the formation of new patches of habitat for plant species able to colonize there (Wang et al., 2008). The change of plant community composition and the maintenance of species diversity as a result of zokor mounds disturbance can be affected mainly from two aspects. The first is that the formation of zokor mounds directly alters the rangeland micro-topography and redistributes soil moisture and temperature, thus affecting the pattern of plant distribution. The second is that zokor mounds lead to a patchy distribution of plants in alpine rangelands. Therefore, we hypothesized that, because of the pattern of zokor mounds disturbance, this mixed patch community is more diverse than would be one with only large community (Begon, Townsend & Harper, 2006). Further, we hypothesized that the changes in the number of zokor mounds could affect plant diversity in alpine rangeland ecosystem.

We selected alpine meadow, alpine steppe and alpine shrub meadow habitats where plateau zokors are typically distributed in the north-eastern of the Tibetan Plateau to study the relationship between zokor mounds and plant diversity. Our goal was to clarify how plateau zokor mounds affect plant diversity in different rangeland types and help deepen our understanding toward co-evolved process, to some extent, provide the local government, farmers and herdsmen with scientific evidence to prevent harm to plateau zokors and conserve plant diversity.

## Materials and Methods

### Study site

**Figure 1:**
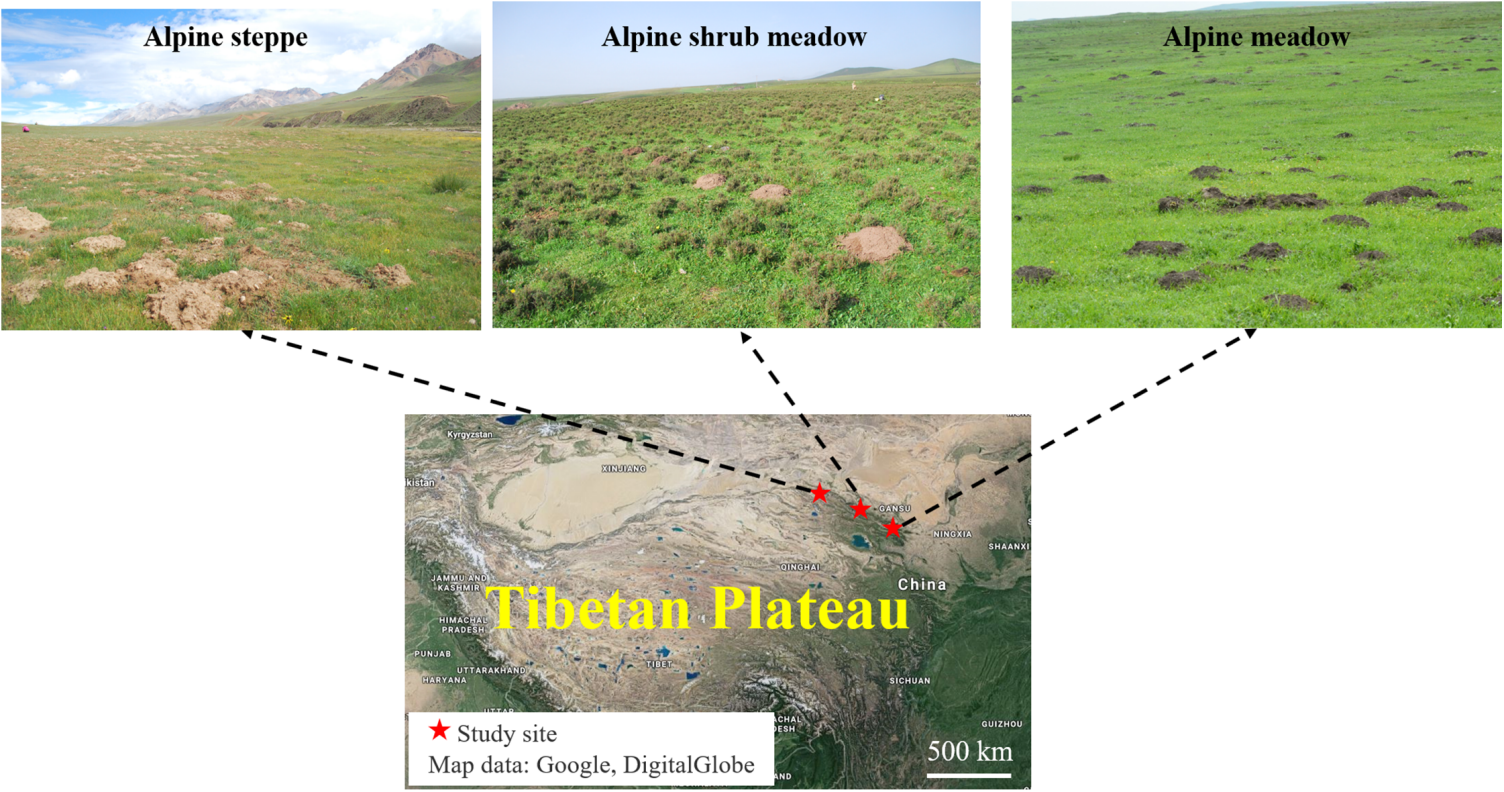
Location of the three major alpine rangeland and the morphological characteristics of zokor mounds. Photos by Yujie Niu. Map data: Google, DigitalGlobe.

The study sites were selected from three representative rangeland types with typical features of plant community inhabited by plateau zokors in the north-eastern of the Tibetan Plateau: alpine meadow, alpine steppe and alpine shrub meadow. These three sites are about 300 km apart from each other (Fig. 1).

The alpine meadow was in Tianzhu Tibetan County at 37°10′33.01″N and 102°49′54.32″E and with an elevation of 2,823 m. The mean annual temperature is −0.6 °C in the past several years, and the mean annual rainfall is 416 mm, which mainly distributes in July–September. The soil is classified as alpine meadow soil. The average diameter of single plateau zokor mound was around 60 cm and the average height was 30 cm, which is largest and highest in the three alpine rangelands. The dominant species in this study site are *Kobresia capillifolia*, *Kobresia humilis*, *Carex lanceolata*, *Elymus nutans*, *Poa pratensis* and *Koeleria cristata*.

The alpine steppe was in Sunan County, Zhangye at 39°30′57.23″N and 98°09′58.57″E and an elevation of 3,458 m above sea level. The mean annual temperature is −1.2 °C in the past several years; the mean annual rainfall is 252 mm. The soil is classified as alpine steppe soil. The average diameter of single plateau zokor mound was around 20 cm and the average height was 10 cm, which is smallest mound in the three alpine rangelands. The dominant species in this study site are *Poa annua*, *E. nutans*, *Stipa capillata* and *Achnatherum splendens*.

The alpine shrub meadow was in Sunan County, Zhangye at 38°46′21.85″N and 99°49′13.15″E and an elevation of 3,189 m above sea level. It has a mean annual temperature of 2 °C and a mean annual rainfall of 350 mm in the past several years. The soil is classified as mountain chestnut soil. The average diameter of single plateau zokor mound was around 40 cm and the average height was 20 cm, which is medium sized mound in the three alpine rangelands. The dominant species in this study site are *Potentilla fruticosa*, *Polygonum viviparum* and *Kobresia capillifolia*. The basic information of these study sites is shown in Table 1.

**Table 1:** The characteristics of study areas on the three alpine rangelands.

Index	Alpine meadow	Alpine steppe	Alpine shrub meadow
Longitude and latitude	N 37°10′33.01″ E 102°49′54.32″	N 39°30′57.23″ E 98°09′58.57″	N 38°46′21.85″ E 99°49′13.15″
Elevation (m)	2,823	3,458	3,189
Soil compaction (kPa)	1,722.09 ± 178.47^a^	688.40 ± 177.74^b^	1,175.93 ± 86.83^b^
Soil moisture (%)	40.68 ± 4.17^b^	23.88 ± 0.92^c^	48.12 ± 1.05^a^
Soil temperature (°C)	13.59 ± 0.66^b^	15.94 ± 0.58^a^	11.95 ± 0.31^c^
Soil pH	7.64 ± 0.03^c^	8.19 ± 0.04^a^	7.90 ± 0.06^b^
Dominant species	*Elymus dahuricus*	*Poa annua*	*Potentilla fruticosa*
	*Kobresia capillifolia*	*Rhodiola rosea*	*Polygonum viviparum*
	*Ruthenian medic*	*Polygonum sibiricum*	*Thalictrum aquilegifolium*

**Note:**

Data are represented as the means ± SE (standard error). Significant differences are denoted by different letters (*P* < 0.05).

### Experimental design

#### Survey of plateau zokor mound density and gradient division

**Figure 2:**
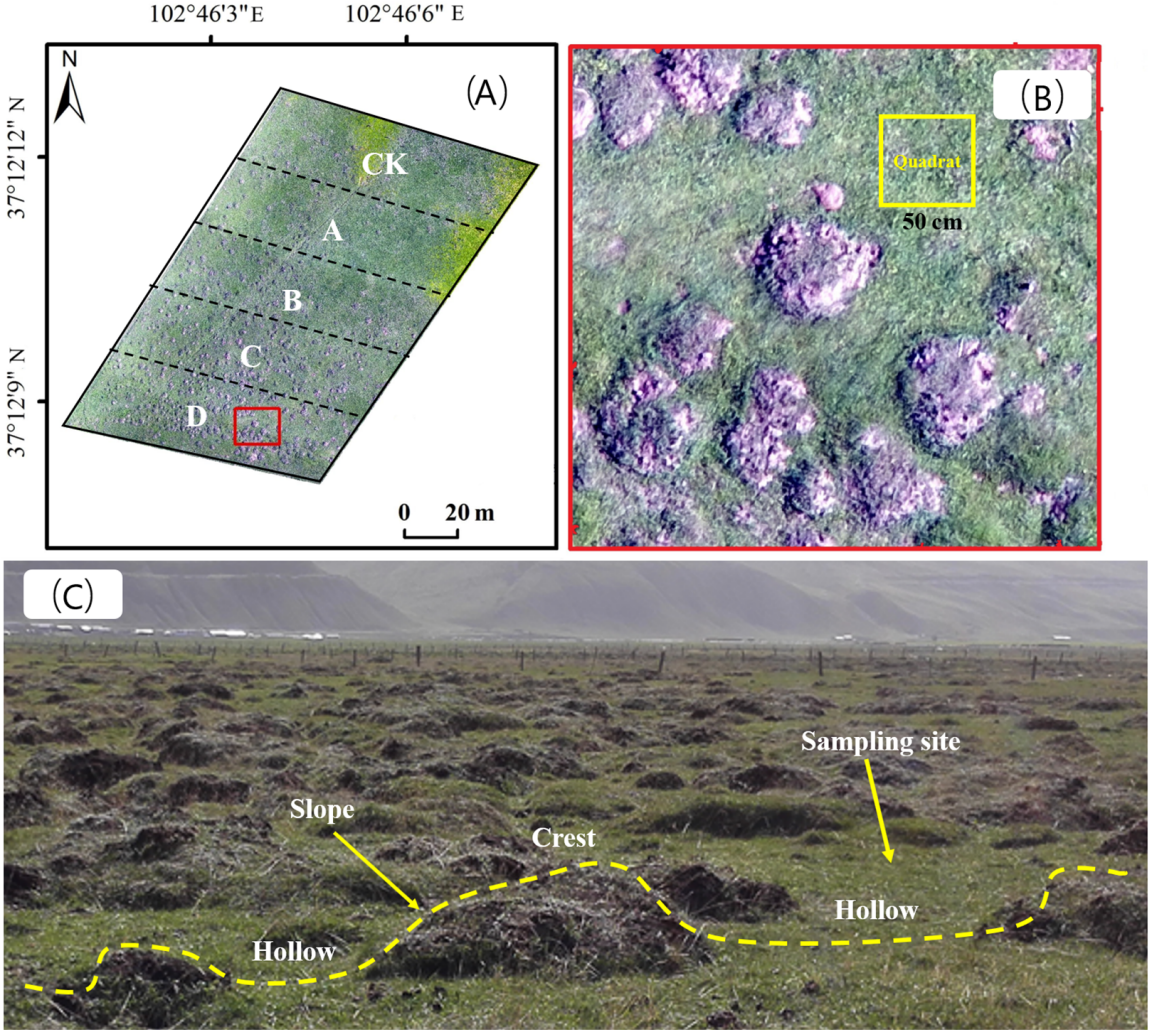
(A) The gradient division of zokor mounds density; (B) sample plot setting and sampling site distribution; (C) the different geomorphologic locations of stabilized rangeland disturbed by plateau zokor. Photos by Yujie Niu.

We consulted technicians at local rangeland stations and herdsmen, and selected areas where plateau zokors are the only single subterranean rodent species with a residence period of more than 10 years. Three replicate gradient plots were selected for each rangeland type, and five adjacent areas with different mound densities were selected from each plot. The area of each density gradient zone was 2,500 m^2^ (Fig. 2A), and the number of new and old plateau zokor mounds in the plots was counted by the artificial counting method. New mounds were those that had recently been formed, within a year, excavation by plateau zokors and without vegetation cover (bare land). Old mounds were more than 2 years old and were covered with vegetation. Mound density was calculated from the total number of old and new mounds. The rangeland without zokor mounds was used as a control (CK), and the plots were classified as plots A, B, C and D with the density of zokor mounds increased. Soil and plant sampling were conducted in non-mound areas, as show in Figs. 2B and 2C. The number of zokor mounds in different plots was show in Table 2. The field work was permitted by College of grassland Science of Gansu Agricultural University.

**Table 2:** The number of zokor mounds in different mounds density plots on the three alpine rangelands.

Plots	Alpine meadow		Alpine steppe		Alpine shrub meadow	
	New mounds	Old mounds	New mounds	Old mounds	New mounds	Old mounds
CK	0.00	0.00	0.00	0.00	0.00	0.00
A	4.67 ± 1.25	15.00 ± 3.74	82.67 ± 10.50	160.67 ± 8.22	16.67 ± 5.31	23.33 ± 4.03
B	14.00 ± 3.74	28.00 ± 6.53	181.33 ± 14.38	173.00 ± 6.48	26.00 ± 4.90	70.67 ± 8.22
C	21.00 ± 2.94	57.67 ± 8.65	331.67 ± 22.69	217.00 ± 12.03	33.00 ± 2.94	124.67 ± 9.53
D	45.00 ± 6.16	103.67 ± 12.92	439.33 ± 28.57	222.00 ± 9.27	83.00 ± 11.43	323.67 ± 18.45

**Note:**

The values represent mean values (±SE).

#### Plant community survey

In each plot, five quadrats were selected at random (Fig. 2B), and we used different sizes of sampling quadrats for the different rangeland types: 0.5 × 0.5 m for alpine meadow, 2 × 2 m for alpine shrub meadow and 1 × 1 m for alpine steppe (Magurran, 1988). The number of species observed in each quadrat was calculated as species richness, and the number of individuals per species in the quadrat was counted as the species abundance. The coverage, height, frequency of each species was also recorded (Niu et al., 2019).

The coverage, abundance, frequency and height of each species in the plant community were standardized by the maximum standardization method.

Species importance value = (relative coverage + relative abundance of species + relative frequency + relative height of species)/4.

The species importance value was used to calculate the diversity of the plant communities in the quadrats (Simpson index, Shannon index) according to the following equations (Magurran, 1988; Niu et al., 2019):

$${\rm{Simpson\ index\!:\ }}D = 1\, - \,\sum\limits_{i - 1}^S {{{\left( {{{{N_i}} \over N}} \right)}^2}} $$

$${\rm{Shannon\ index\!: }}\ H\, = \, - \,\sum\limits_{i = 1}^S {{{{N_i}} \over N}} \left( {\ln {{{N_i}} \over N}} \right)$$

where *S* is the total number of species in each quadrat; *N* is the sum of the relative importance values of all *S* species; *N_i_* is the relative importance value of the *i*-th species; and *N*_max_ is the maximum relative importance value.

#### Soil property survey

In each mound density zone, we investigated the soil moisture, temperature at depths of 0–10, 10–20 and 20–30 cm close to the plant quadrats. Soil moisture and temperature were measured using a Top TZS-IIW device (Zhejiang Top Instrument Co., Ltd., Hangzhou, China) within three replications.

### Data analysis

In the three alpine rangelands, we first analyzed changes in plant diversity and soil moisture and temperature in relation to the different mound densities. Multiple comparisons were made for species richness, abundance, Simpson, Shannon, soil temperature and moisture between different mound density zones in different rangeland types using the least significant difference method. The cumulative species richness was compared between different mound densities using species accumulation curves, and the species abundance distribution within the community was assessed by the species abundance rank. Plant species were classified into five functional groups (grasses, sedges, perennial forbs, annual forbs and shrubs, Klein, Harte & Zhao, 2007), and the functional group data were expressed as the sum of the importance values of the different species within the functional group. Changes in the functional groups with increasing zokor mound density were analyzed in different alpine rangelands. Finally, a structural equation model was constructed to quantify the direct and indirect effects of zokor mounds and soil moisture and temperature on plant diversity.

#### Plant cumulative species richness

The species accumulation curve is a graph recording the cumulative species richness of living things recorded in a particular environment as a function of the cumulative effort expended searching for them (Willott, 2001). The rate at which new species are added to the inventory provides important clues about the species richness, and indeed the species abundance distribution, of the assemblage as a whole (Magurran, 1988). The species richness in the sample plots was denoted SR_P_. The cumulative species richness in each plot was calculated using Jack 1: SR (*J*) = SR (ob) + *a ×* *n*/(*n−*1), where SR (ob) is the observed actual species richness value in one quadrat; *a* is the number of species that appear in one quadrat; and *n* is the number of quadrats (Dove & Cribb, 2006).

#### Species abundance distribution

Based on the species abundance in a plant community, species were ranked in descending order with increasing mound density for each density zone in the three rangeland types. Species abundance distribution can be best visualized in the form of relative abundance distribution plots. The geometric model was used for fitting the alpine steppe, because the geometric model fits observed species abundances in highly uneven communities with low diversity. This is expected to occur in terrestrial plant communities (as these assemblages often show strong dominance) as well as communities in harsh environments (Fattorini et al., 2016; Magurran, 1988). Because there is more species in the alpine meadow and alpine shrub meadow with relatively even distribution. The logseries model was used for fitting the alpine meadow and alpine shrub meadow (Magurran, 1988). The log series produces a slightly more even distribution of species abundances than the geometric series. The goodness of fit of the model was estimated using *R*^2^.

#### Structural equation model

We constructed a structural equation model for the path relationship between three latent variables: zokor mounds, soil moisture and temperature and plant diversity. The observable variables for zokor mounds were the number of new mounds and the number of old mounds, and the observable variables for soil moisture and temperature were soil temperature and soil moisture. The observable variables for plant diversity were richness and abundance as well as the Simpson, Shannon, evenness and Berger–Parker indices. The path coefficients and parameters were estimated using the maximum likelihood method, and the goodness of fit of the model was estimated using *χ*^2^, GFI and RMSEA (Ma et al., 2017).

Statistical analysis was conducted using SPSS 19.0. The significance threshold was pre-established as α = 0.05. The species accumulation curve was constructed using Estimates Win910. The species abundance distribution was constructed using the software Past 3. Graphs were drawn using Origin 9.0, and the structural equation model was constructed using AMOS 17.0.

## Results

### Effect of plateau zokor mounds on plant diversity in the three rangelands

**Figure 3:**
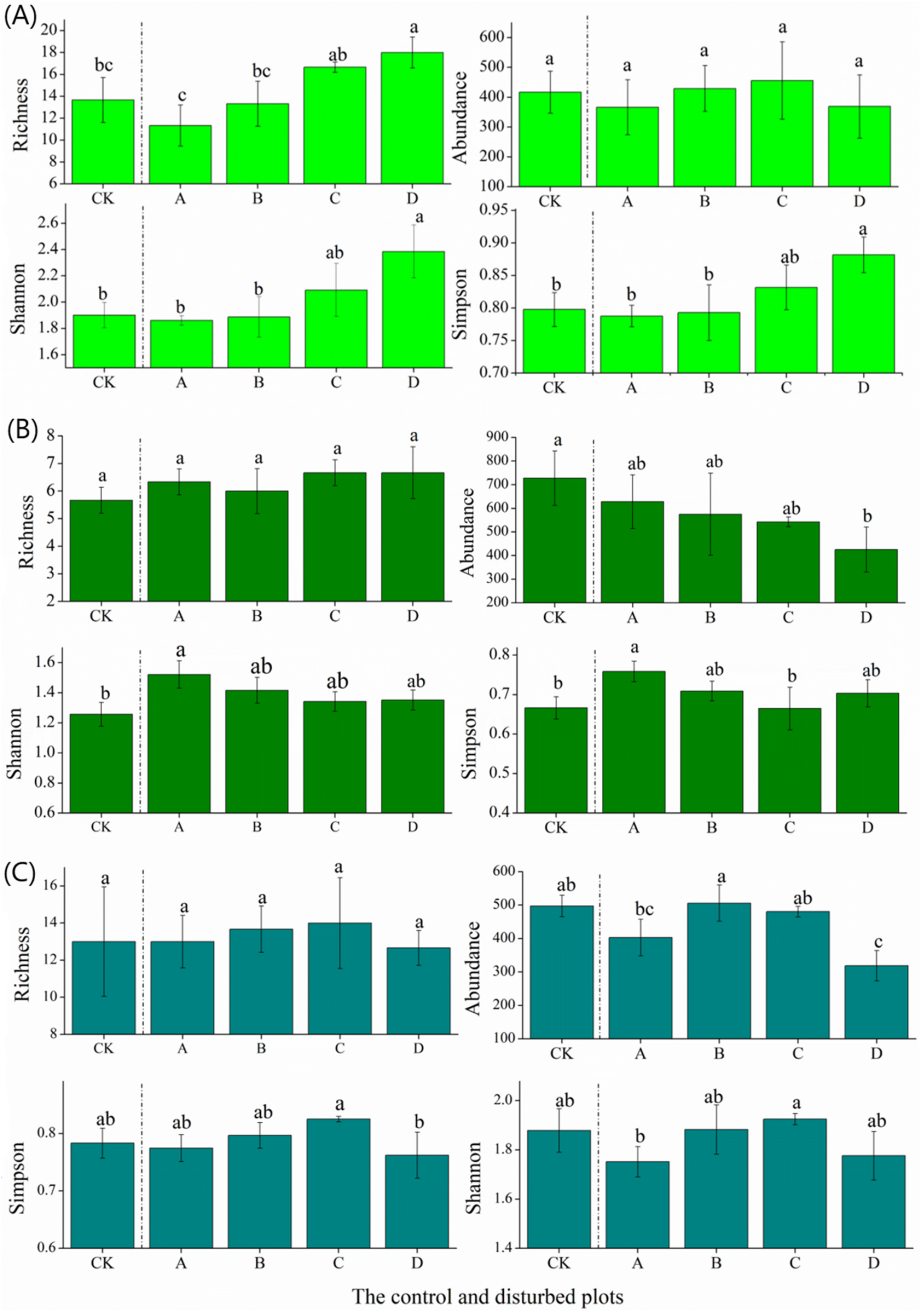
Effect of zokor mounds on plant diversity in the alpine rangeland. (A) Alpine meadow; (B) alpine steppe; (C) alpine shrub meadow.

In the alpine meadow, the species richness and the Shannon and Simpson indices of the plant communities increased significantly with the increasing density of the plateau zokor mounds. The species abundance was not affected by mound density (Fig. 3). In the alpine steppe, the species richness and Shannon and Simpson indices of the plant communities decreased significantly with increasing mound density (Fig. 3). In the alpine shrub meadow, the species richness and Shannon and Simpson indices of the plant communities in non-mound areas first increased and then decreased with increasing mound density. There was no significant relationship between species richness and mound density (Fig. 3).

### Effect of plateau zokor mounds on soil temperature and moisture in three rangelands

**Figure 4:**
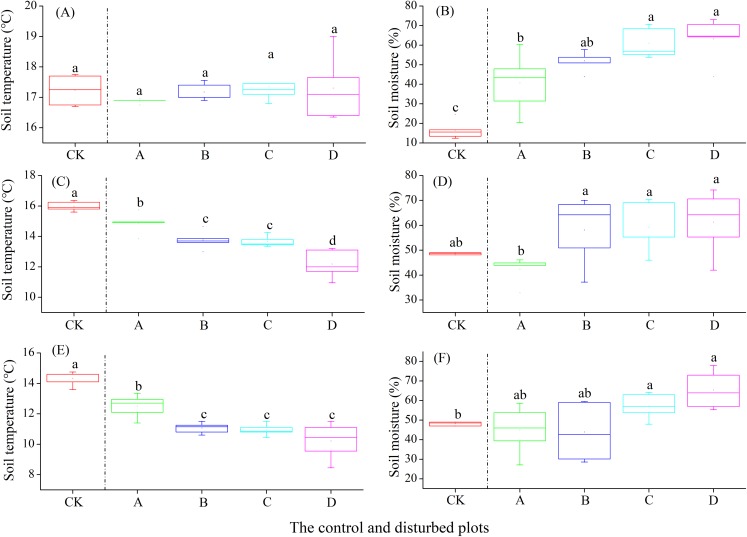
Effect of zokor mounds on soil temperature and moisture in the alpine meadow. (A–B) 0–10 cm; (C–D) 10–20 cm; (E–F) 20–30 cm.

In the alpine meadow, soil temperature at the 0–10 cm layer did not change significantly with increasing plateau zokor mound density, whereas there were significant decreases at the soil layer of 10–20 and 20–30 cm. Meanwhile, soil moisture increased significantly at the soil layer of 0–10, 10–20 and 20–30 cm (Fig. 4).

**Figure 5:**
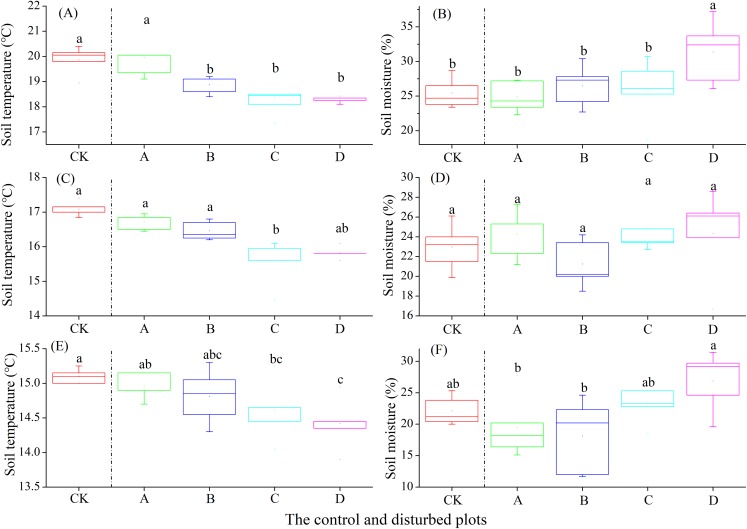
Effect of zokor mounds on soil temperature and moisture in the alpine steppe. (A–B) 0–10 cm; (C–D) 10–20 cm; (E–F) 20–30 cm.

In the alpine steppe, the soil temperature at the depths of 0–10, 10–20 and 20–30 cm decreased significantly with increasing density of plateau zokor mounds. In contrast, soil moisture at the depths of 0–10 and 20–30 cm increased significantly, but no significant change occurred at the depth of 10–20 cm (Fig. 5).

**Figure 6:**
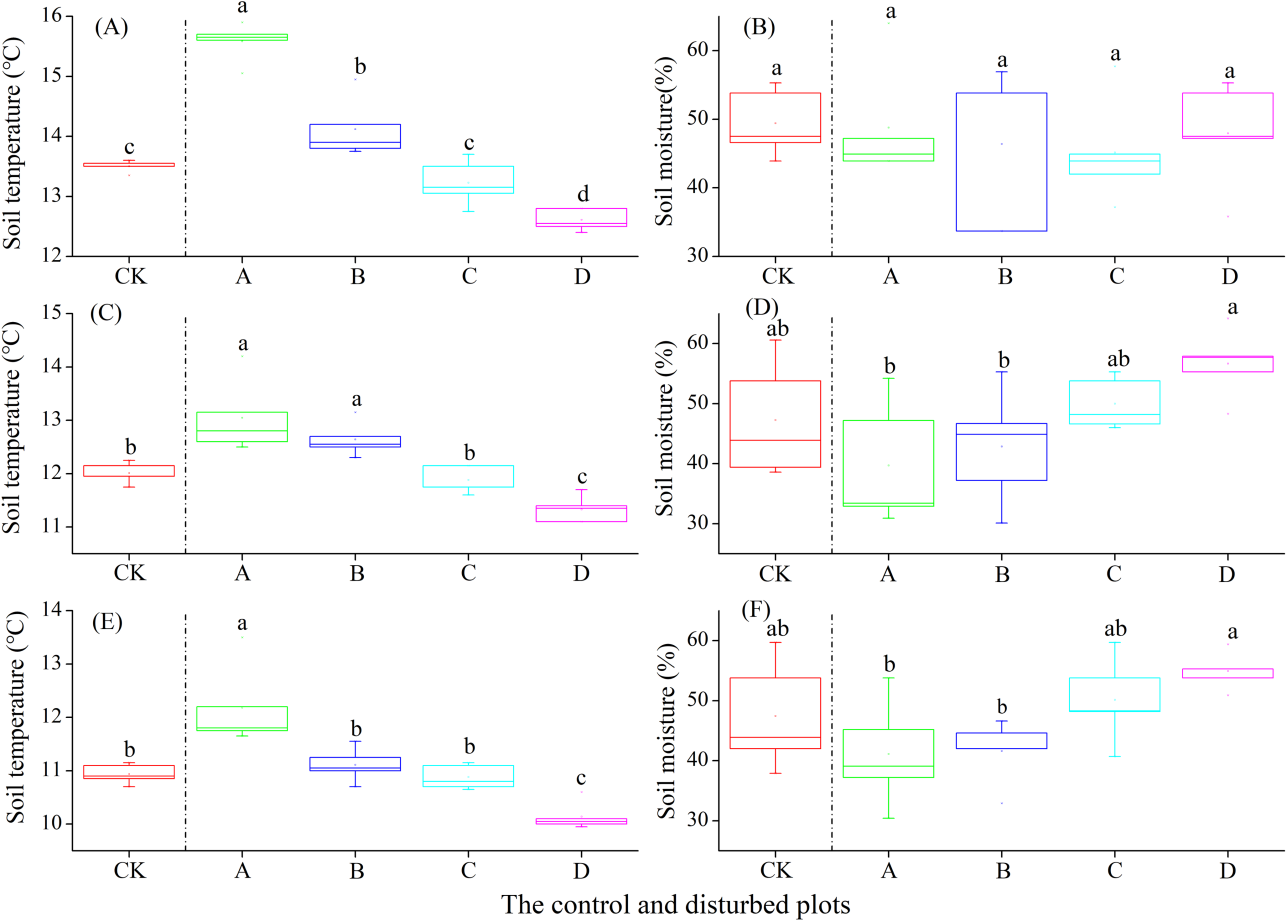
Effect of zokor mounds on soil temperature and moisture in the alpine shrub meadow. (A–B) 0–10 cm; (C–D) 10–20 cm; (E–F) 20–30 cm.

In the alpine shrub meadow, the soil temperature at the depths of 0–10, 10–20 and 20–30 cm decreased significantly with the increasing density of plateau zokor mounds. In contrast, the soil moisture at the depths of 10–20 and 20–30 cm increased significantly, but there was no significant change in soil moisture at the depth of 0–10 cm (Fig. 6).

### Changes in cumulative species richness with different mound densities and abundance distribution in the three rangelands

**Figure 7:**
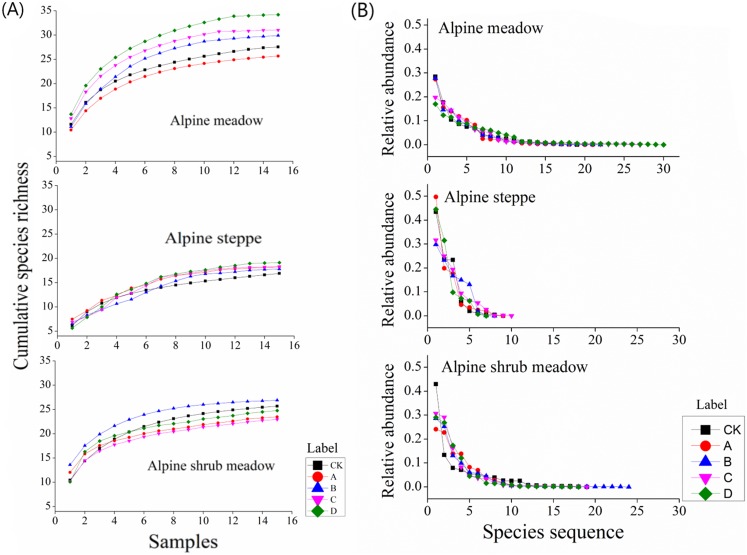
(A) Changes in cumulative species richness with increasing mound density and (B) plant abundance distribution in the three rangelands.

Figure 7A shows the accumulation of species richness with different mound densities in the three rangeland types. In the alpine meadow, the cumulative species richness and the accumulation rate were increased with increasing mound density. In the alpine steppe, the above two parameters first decreased and then increased with increasing mound density. In the alpine shrub meadow, these two parameters initially increased and then decreased with increasing mound density.

In the alpine meadow, all the species abundance distribution in the alpine meadow could be fitted by the logseries model with *R*^2^ > 0.93. Furthermore, species abundance distribution was relatively even. In the alpine steppe, the fitted curves were relatively steep. All the species abundance distribution in the alpine steppe could also be fitted by the geometric model with *R*^2^ > 0.79. In the alpine shrub meadow, the fitted curves were also relatively even. All the species abundance distribution in the alpine shrub meadow could also be fitted by the logseries model with *R*^2^ > 0.87 (Fig. 7B). Above all, the species number that predominantly structure the plant community was fewer in the alpine steppe than alpine shrub meadow and alpine meadow.

### Changes in functional group composition with different mound densities in the three rangelands

**Figure 8:**
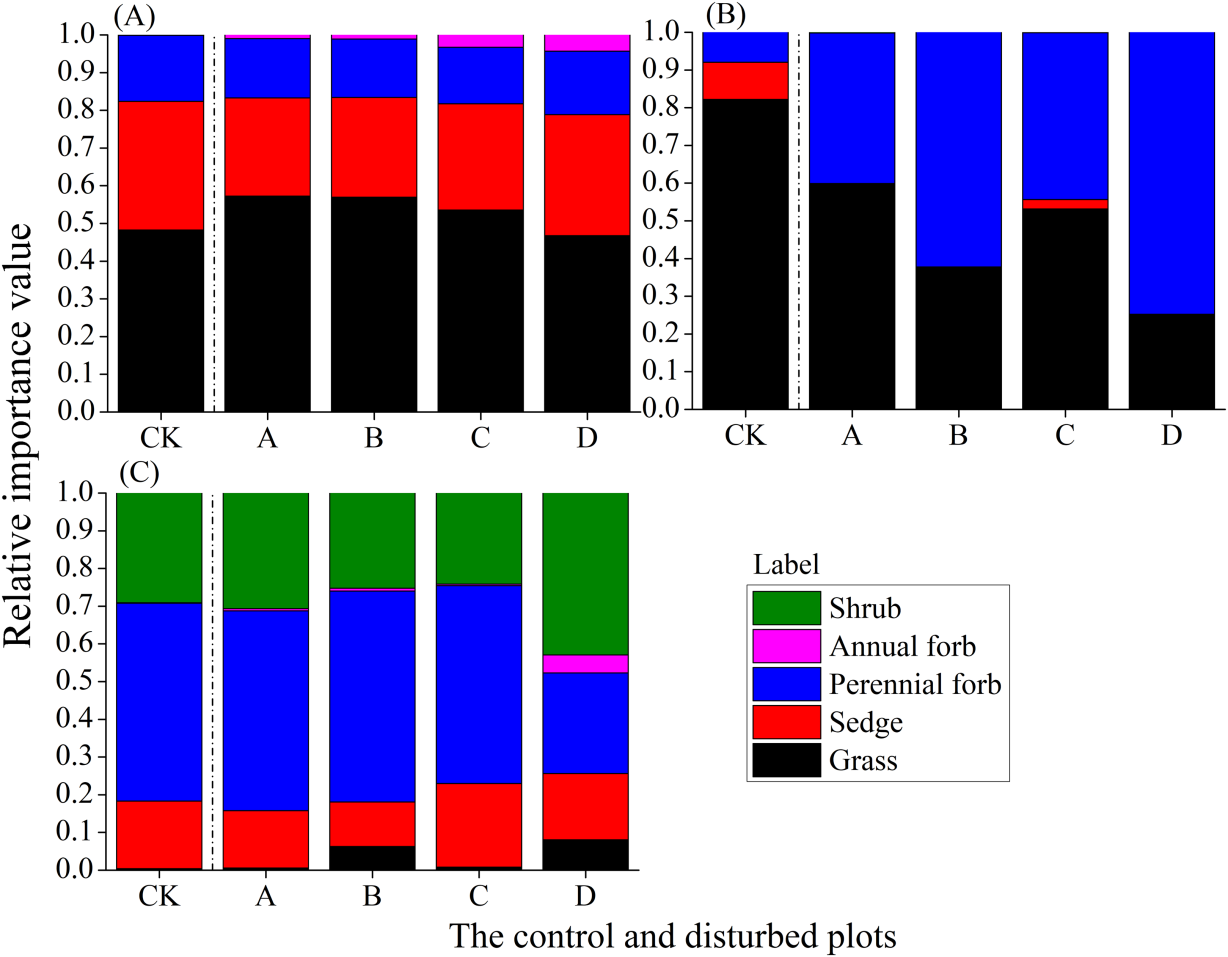
Changes in the relative importance values of plant functional groups with increasing mound density in the three alpine rangelands. (A) Alpine meadow; (B) alpine steppe; (C) alpine shrub meadow.

In the alpine meadow, the proportion of grasses decreased with increasing mound density, while the proportions of sedges, perennial forbs and annual forbs increased; annual forbs showed the highest rate of increase (Fig. 8A). In the alpine steppe, there was no clear trend of change in the proportion of different functional groups with increasing mound density. However, the proportion of perennial forbs increased (Fig. 8B). In the alpine shrub meadow, the proportion of shrubs first decreased and then increased with increasing mound density, while the proportion of perennial forbs first increased and then decreased (Fig. 8C).

### The effects of zokor mounds to soil properties and plant diversity in the three rangelands

**Figure 9:**
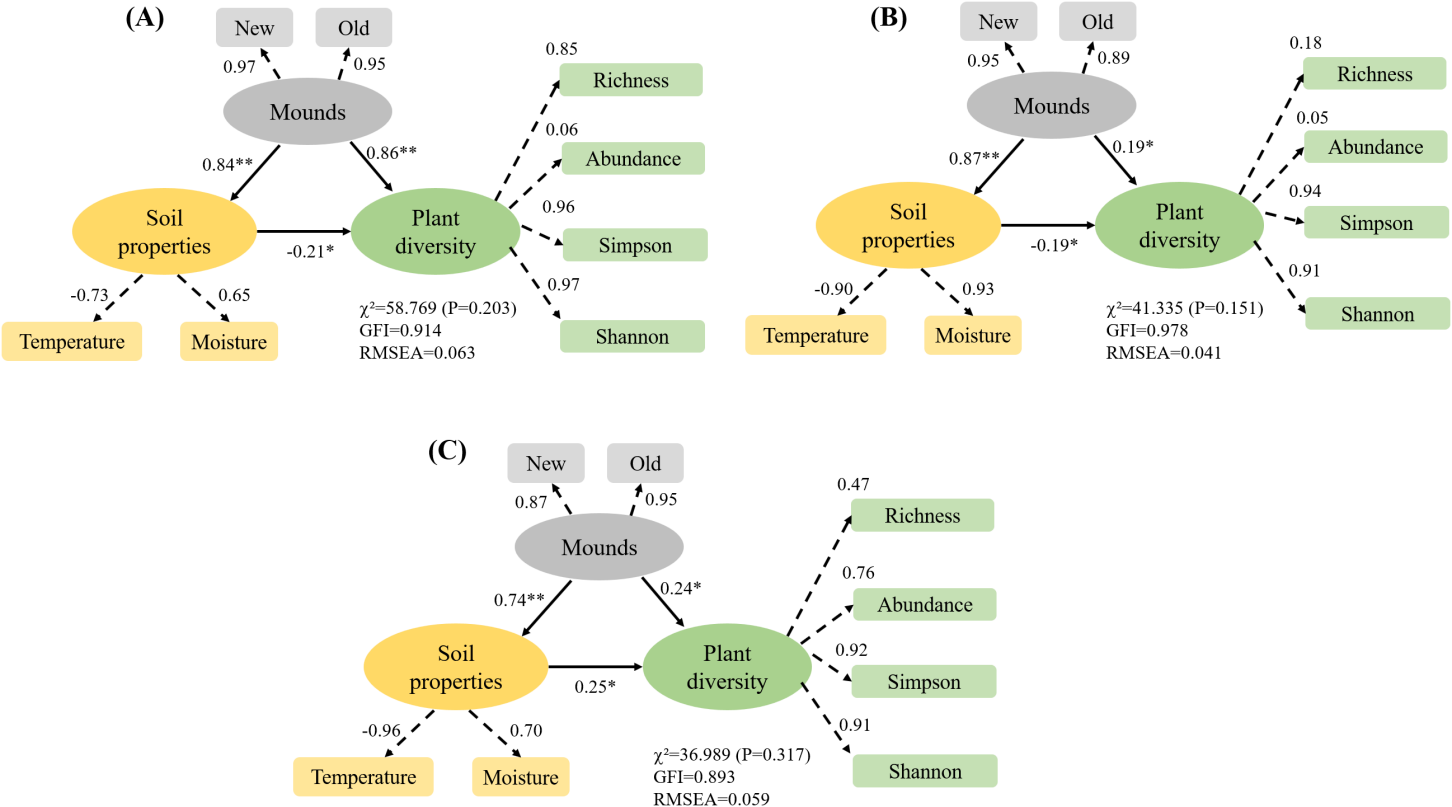
The effects of zokor mounds and soil properties on plant diversity. *P < 0.05 and **P < 0.01. (A) Alpine meadow; (B) alpine steppe; (C) alpine shrub meadow.

In the alpine meadow, the direct effect of zokor mounds on soil properties was 0.84, and the direct effect on plant diversity was 0.86 (Fig. 9A). In the alpine steppe, the direct effect of zokor mounds on soil properties was 0.87, and the direct effect on plant diversity was 0.17 (Fig. 9B). In the alpine shrub meadow, the direct effect of zokor mounds on soil properties was 0.74, and the direct effect on plant diversity was 0.24 (Fig. 9C). Soil moisture and temperature was significantly affected by zokor mounds. Among the three rangeland types, zokor mounds had the greatest effect on plant diversity in the alpine meadow.

## Discussion

### The indirect effects of zokor mounds on plant diversity are directly realized through creating greater spatial heterogeneity

Subterranean rodents, one of the most important components of rangeland ecosystems, are regarded as ecosystem engineers that greatly influence ecological processes such as soil nutrient cycling, spatial heterogeneity and plant diversity (Hu et al., 2017; Reichman & Seabloom, 2002; Smith & Foggin, 1999; Zhang, Zhang & Liu, 2003). Subterranean rodents show different degrees of importance and irreplaceability compared with aboveground herbivores. Mielke (1977) believed that subterranean rodents are the most direct drivers of change in soil chemical properties in North American rangelands and that their underground excavation activity is important for soil nutrient cycling. Grinnell (1932) concluded that plant diversity would rapidly decline in the region if populations of subterranean rodents went extinct. Subterranean rodents dig burrows and push soil toward the ground surface to form mounds, this is the most direct and obvious effect of such animals on soil and plant communities (Smith & Foggin, 1999; Thorn, 1978). Mound creation considerably changes the microtopographic features of the land surface and leads to the redistribution of soil temperature and moisture. Because mounds are significantly higher than the surrounding non-mound area, rainwater accumulates in the low-lying area. Additionally, the mounds change the light conditions in the surrounding area to some extent, thereby changing soil temperature. Plateau zokors, a unique rodent species that lives underground on the Tibetan Plateau, are mainly distributed in alpine meadow, alpine shrub meadow and alpine steppe areas (Cai et al., 2018; Zhang, Zhang & Liu, 2003). There is no scientific basis for that plateau zokors compete with livestock for food resources and cause soil erosion and rangeland degradation. The local government and herdsmen organize to eradicate zokors every year and hope to completely eliminate this species (Zhang, Zhang & Liu, 2003). But the animals are capable of changing the physical conditions of their living environment and thus created high heterogeneity environment, and then strongly affect the distribution of other organisms. Environments with high heterogeneity created an ecological condition where presenting a wider range of niches, and more species as a consequence. The results of this study showed that the soil temperature in non-mound areas decreased with increasing mound density, while the soil moisture increased in the three rangeland types on the Tibetan Plateau, indicating that the formation of mounds by plateau zokors led to the redistribution of soil moisture and temperature. Hu et al. (2017) also concluded that plateau zokors had greatly direct impact on the soil physiochemical properties through their foraging and burrowing activities. Wang et al. (2008) found that zokor disturbance resulted in a significant change in soil particle size, increased soil water content from 20% to 25%. In this study, structural equation modeling also revealed highly significant effects of mounds on soil moisture and temperature and then on plant diversity in the three rangeland types (Fig. 9). This suggests that the indirect effects of zokor mounds on plant diversity are directly realized through creating greater spatial heterogeneity (such as soil temperature and moisture).

### The direct effects of zokor mounds on plant diversity maybe realized through cyclic succession on bare mound patch

Plateau zokor mound is a common disturbance in the alpine rangeland ecosystem (Hu et al., 2017; Wang et al., 2008; Yang et al., 2017; Zhang et al., 2012). Zokor mounds make the plant communities in alpine rangelands spatially heterogeneous and dynamic mosaic communities. Our study showed that the plant diversity increased significantly in the alpine meadow and decreased significantly in the alpine steppe with increasing mound density, but it initially increased and then decreased in the alpine shrub meadow. The results of the structural equation model showed that zokor mounds directly affected plant diversity significantly in different types of alpine rangeland on the Tibetan Plateau. The plant diversity showed different trends of change with increasing mound density among the different rangeland types, and the trends of change in a plant community as a result of a zokor mounds disturbance may be explained mainly by a direct mechanism. That is, within short periods, new zokor mound that randomly generated undergo secondary succession in local areas. These new mounds eventually form stable communities that are mosaics of different types of communities. There is thus a cyclic series from bare mounds to climax community then to bare mounds. Yang et al. (2017) also concluded that there was much variation in species composition at earlier stages of succession on the bare mounds, but communities on older mounds became more similar to each other and to their surrounding vegetation over the course of secondary succession. The alpine steppe is a fragile rangeland ecosystem with a thin soil layer and low soil nutrients, and low soil moisture and low plant density. Owing to the harsh environmental conditions, the species abundance distribution curve was steepest for the plant community, indicating that plant communities were dominated by the dominant species in this ecosystem which resulted in low species richness. Because the non-mound areas are considered dynamic mosaic plant communities resulting from succession toward a stable state from bare mound patches formed in different periods, the current climax community have undergone multiple succession processes from bare mound patches. During each cyclic succession, the dominant species become more abundant, thus reducing the diversity of the alpine steppe and even causing irreversible degeneration. Therefore, the control and prevention of zokor should be strengthened in the alpine steppe. In contrast, the alpine meadow and alpine shrub meadow have better environmental conditions, including a thicker soil layer and higher soil nutrient contents as well as a high density of vegetation that can be rapidly restored. Thus, these two ecosystems can resist a certain degree of disturbance from zokor mounds. In these two ecosystems, the abundance distribution curve of the community was relatively flat. Because the available resources for plants are abundant, the interspecific competition in the plant communities is relatively low. Due to the dynamic mosaic of plant communities, each plant species has an equal opportunity to colonize bare mound patches during the succession process, so the climax community accommodate richer plant communities in the alpine meadow. Because mixed small communities are more diverse than single large communities (Begon, Townsend & Harper, 2006), so the alpine meadow has higher plant diversity. Moreover, the cumulative species richness also increased with increasing mound density in the alpine meadow (Fig. 7), further indicating that zokor mounds increased the heterogeneity of plant communities. This study also resulted that “opportunistic” annual plants increased the fastest with increasing mound density in the alpine meadow (Fig. 8). Plant diversity showed unimodal changes with increasing mound density in the alpine shrub meadow, and this may be because the alpine shrub meadow contains higher soil moisture per se, while the formation of zokor mounds further increases soil moisture. This could limit some mesophytic and xenobiotic plants, so plant diversity would initially increase and then decrease with increasing mound density. The possible mechanisms by which zokor mounds directly affect plant diversity may be explained by the fact that the time and potential of cyclic succession on the bare mound patches are different.

### Zokor mounds disturbance on alpine rangeland can deepen our understanding toward co-evolved process

**Figure 10:**
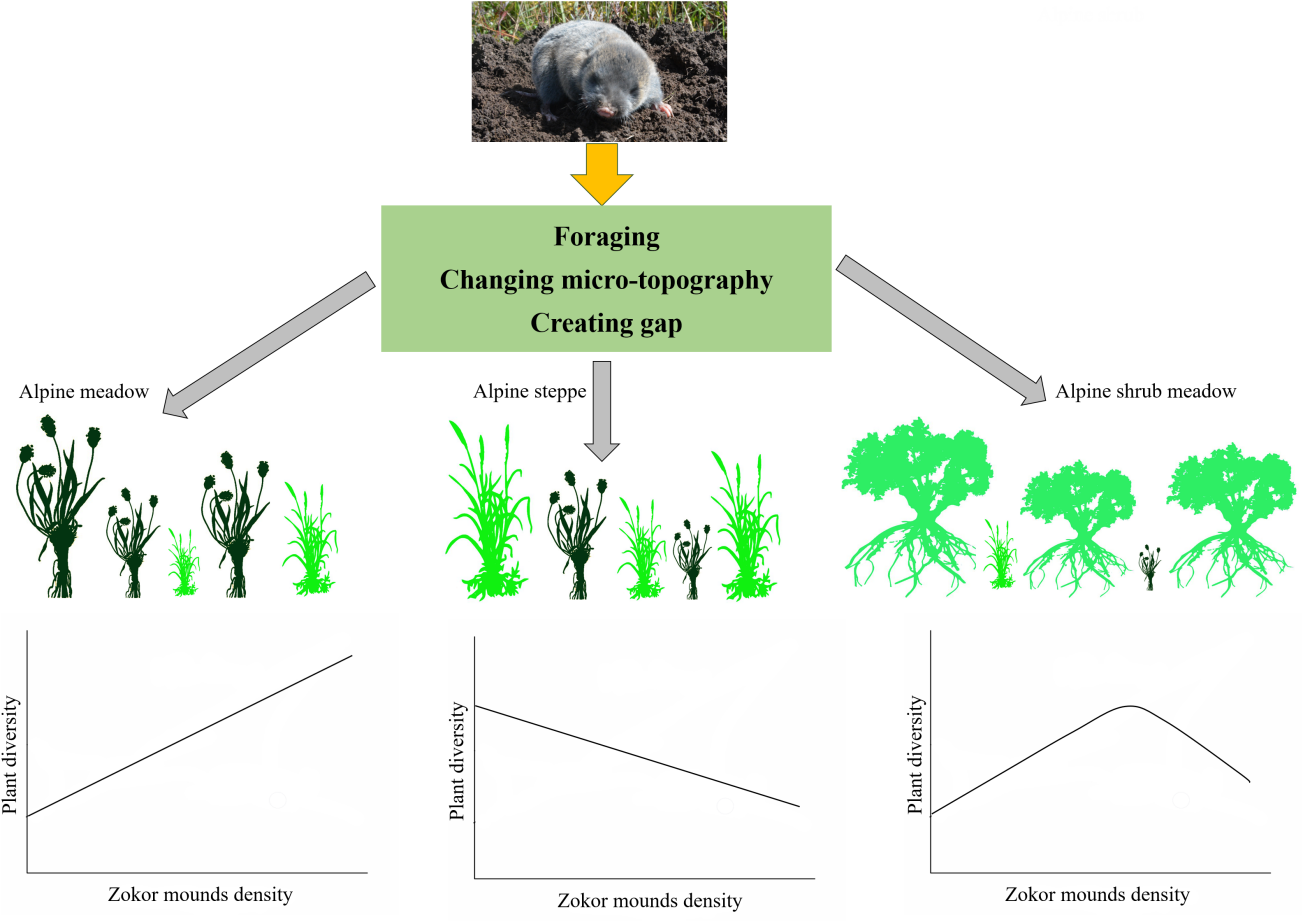
Summary diagram of zokor affect plant diversity in three alpine rangelands. Photos by Yujie Niu.

Disturbances constituting a series of events play a significant role in the structure and function of ecosystems (Battisti, Poeta & Fanelli, 2016c; Dornelas, 2010; Mackey & Currie, 2001; Sousa, 1984). A first substantial distinction of disturbance typologies can be classified into biotic disturbances and abiotic disturbances. Disturbances caused by animals are usual in various natural environments and are associated to the different functions carried out by living organisms over their daily, seasonal, annual and life cycles (Brawn, Robinson & Thompson, 2001; Gibson, 1989; Sousa, 1984). Such disturbances can be classified into five popular types at least: trampling, foraging, tunnel digging, feces and urine deposition (Battisti, Poeta & Fanelli, 2016a). Due to the year-round subterranean life of some rodents, so there are no trampling effects. The disturbances induced from animal can be recognized as significant agents of natural selection. They play a very important role in changing the structure, composition, diversity, distribution and the long-term co-evolution dynamics of plant and animal (Dornelas, 2010). Herbivorous and subterranean rodents are widely distributed around the rangeland regions of the earth except two continents (Australia and Antarctica) (Andersen, 1987). The plateau zokors, which are the only small herbivorous subterranean mammal on the Tibetan plateau, may have great effects on the alpine ecosystem in different kinds of ways because of their specific behavioral pattern, lifestyle and population dynamic (Wang et al., 2008; Yang et al., 2017). In China, plateau zokors have been conventionally regarded as pests and elimination projects have been implemented by local governments because they competed with cattle, and caused soil erosion to a certain degree (Smith & Foggin, 1999; Zhang, Zhang & Liu, 2003). The change of plant community composition and the maintenance of species diversity in alpine rangeland as a result of zokor mounds disturbance can be explained mainly from three aspects (Fig. 10). Tunnel digging is the main activity performed by alpine zokor to affect ecosystems, resulting in the formation of micro-topography and gap. The foraging of zokor belongs to below-ground foraging; it is difficult to quantitatively evaluate the impact of zokors foraging on the alpine rangeland. So, we focused attention on the tunnel digging and mound making activities. Future work should quantitatively study the zokor foraging by using stable isotope techniques. We can then evaluate the effects of zokor foraging to rangeland ecosystem. We emphasize the key role of plateau zokors play on the rangeland ecosystem of Tibetan plateau. We reckon plateau zokor to be a keystone species because: (1) it makes mounds that are the primary habitat to a great range of niches for plants living; (2) it creates micro-topography that results in the increasing of plant micro-patches; (3) it will be conducive to plant community and ecosystem-level dynamics; (4) underground excavation activity is important for soil nutrient cycling. But disturbance by zokor mounds has different effects on plant diversity in different ecosystems and the anti-interference ability of the Tibetan Plateau. Zokor mounds lower the species diversity of plant communities in the fragile alpine steppe while laying a foundation for the co-existence of various species in plant communities and the maintenance of plant diversity under favorable alpine ecosystem (Fig. 10). In order to ensure the lasting sustainable use of alpine rangeland on Tibetan Plateau accompanied by the equilibrium of ecosystem and conservation of native biological diversity, plateau zokors should be managed jointly with other integrated management approach on the alpine rangeland ecosystem.

## Conclusions

In China, plateau zokors have been considered a negative element (pest), competitors with cattle and agents of soil erosion; thus, there are widespread mechanical or poisoning activities to kill zokors. But the results of this study reveal that the soil temperature decreased significantly, and the soil moisture significantly increased, with the mound density increasing in all three rangelands. Furthermore, plant diversity is closely related to the density of zokor mounds in three alpine rangelands. Plateau zokor mounds dominated the distribution of soil moisture and temperature and significantly affected plant diversity, that further deepens our understanding toward co-evolved process. We think plateau zokor to be a keystone species on the Tibetan plateau. We argue about that blindly killing or poisoning the plateau zokor is destructive to the conservation of native biological diversity and the natural function of the rangeland ecosystem. In addition, we highly suggest that large-scale poisonings of plateau zokor should be halted.

## Supplemental Information

10.7717/peerj.6921/supp-1 Supplemental Information 1 The raw data of different figures.

10.7717/peerj.6921/supp-2 Supplemental Information 2 (a). Rodenticides are blanketly used to kill the plateau zokor and pika (a small rabbit-like mammal) on the Tibetan Plateau. (b). The figure shows the “fruits” of killing plateau zokor. Photos by Yujie Niu.

## Additional Information and Declarations

### Field Study Permissions

The following information was supplied relating to field study approvals (i.e., approving body and any reference numbers): Field work on three rangeland types in the north-eastern part of the Tibetan Plateau was permitted by College of Grassland Science of Gansu Agricultural University.

### Data Availability

The following information was supplied regarding data availability: The raw data are available in the Supplemental File.
