# Supplementary figures and images for: Plant diversity is closely related to the density of zokor mounds in three alpine rangelands on the Tibetan Plateau
Source: PeerJ. 2019 May 13;7:e6921. doi: 10.7717/peerj.6921 (PMC6521815; doi:10.7717/peerj.6921)

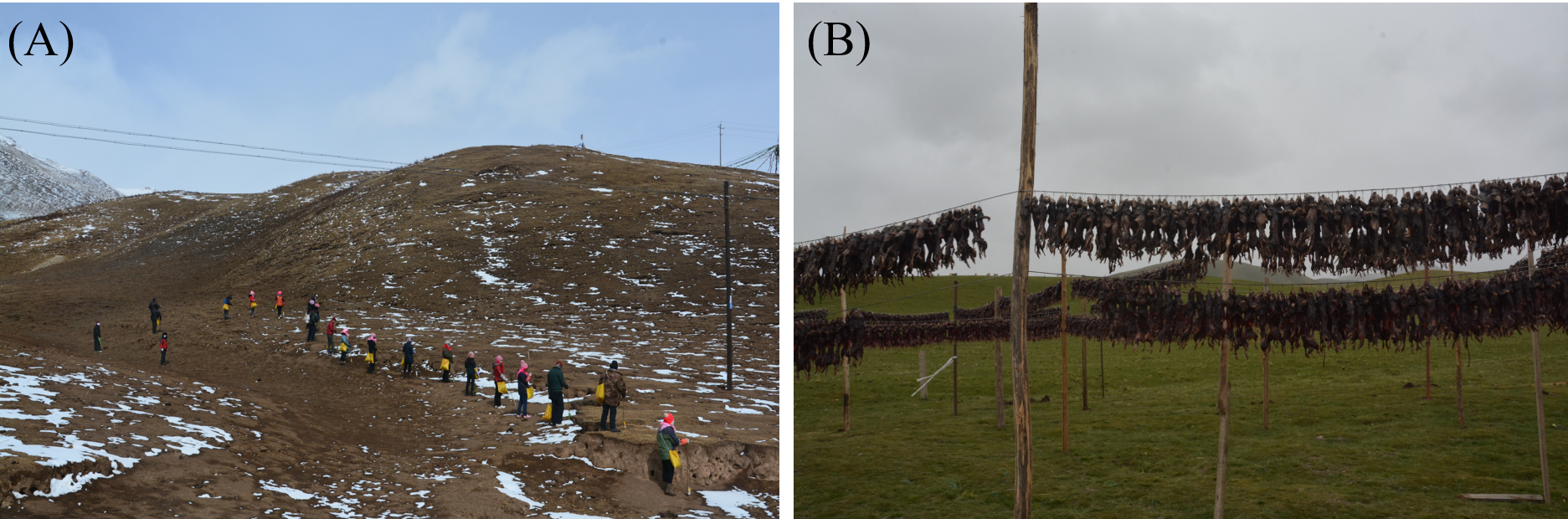

Supplement: Supplemental Information 2 — Photos by Yujie Niu. [file peerj-07-6921-s002.png]
